# Supplementary material for: Content-rich biological network constructed by mining PubMed abstracts
Source: BMC Bioinformatics. 2004 Oct 8;5:147. doi: 10.1186/1471-2105-5-147 (PMC528731; doi:10.1186/1471-2105-5-147)
Supplement: Additional File 2 — The original results of the above study (non-essential files are deleted to keep the file size under the limit set by BMC bioinformatics). [file 1471-2105-5-147-S2.bz2 › chilibotAdditionalFile2/dip40/705ID10734142E18042/html/MUTH_MUTS.html]

 


 **MUTH** and **MUTS** 
  
Found 88 abstracts in PubMed, retrieved 40.  
 

 What does Google say? 
 PDF only 
| .edu only 

---

**Interactive relationship** (e.g. stimulation, inhibition, etc)

**Neutral relationship**- These results suggested that depletion of  **MutS**  in nutritionally stressed cells was possibly caused by the relative instability of  **MutS**  compared with MutL and  **MutH** .  Ref: 8636043 J Bacteriol, 1996
- Protein affinity chromatography experiments confirmed that MutL, but not  **MutS** , physically associates with  **MutH** .  Ref: 9880500 J Biol Chem, 1999
- Activation of  **MutH**  requires the recognition of a DNA mismatch by  **MutS**  and MutL.  Ref: 9482749 EMBO J, 1998
- An unsolved problem in E. coli mismatch repair is how the  **MutS**  MutL complex communicates positional information of a mismatch to  **MutH** .  Ref: 11888282 Biochemistry, 2002
- These results suggest that a  **MutS**  MutL complex in the vicinity of a mismatch is involved in activating  **MutH** .  Ref: 11371566 J Biol Chem, 2001
- This helix appears to act as a molecular lever through which  **MutS**  and MutL may communicate the detection of a DNA mismatch and activate  **MutH** .  Ref: 9482749 EMBO J, 1998
- Recent crystallographic and biochemical studies have revealed that MutL operates as a molecular switch with its interactions with  **MutH**  and  **MutS**  regulated by ATP binding and hydrolysis.  Ref: 10946232 Mutat Res, 2000
- All possible pairwise combinations of UvrD, MutL,  **MutS** , and  **MutH**  were tested using the yeast two hybrid system to identify potential interactions involving mismatch repair proteins.  Ref: 9880500 J Biol Chem, 1999
- The T.thermophilus  **mutS**  gene complemented the hypermutability of the E.coli  **mutS**  mutant, suggesting that T.thermophilus  **MutS**  protein was active in E.coli and could interact with E.coli MutL and or  **MutH**  proteins.  Ref: 8604304 Nucleic Acids Res, 1996
- **MutS**  initiates repair by binding to the mismatch, and activates together with MutL the  **MutH**  endonuclease, which incises at hemimethylated dam sites and thereby mediates strand discrimination.  Ref: 11920679 J Cell Physiol, 2002
- To initiate mismatch repair in Escherichia coli, three proteins are essential,  **MutS** , for mismatch recognition,  **MutH** , for introduction of a nick in the target strand, and MutL, for mediating the interactions between  **MutH**  and  **MutS** .  Ref: 10946232 Mutat Res, 2000
- Such a higher order  **MutS**  complex productively interacts with MutL in ATP hydrolyzing conditions and generates a specific ternary complex, which might communicate with  **MutH** .  Ref: 11888282 Biochemistry, 2002

**Non-interactive relationship** (e.g. studied together, co-existance, homology, etc.)

- Consequently, we tested whether the amounts of  **MutS** , MutL, and  **MutH**  were regulated by two global regulators, RpoS sigma38 and Hfq HF I putative RNA chaperone, which are involved in stationary phase transition.  Ref: 9393714 J Bacteriol, 1997
- In mut umuC122 Tn5 bacteria, UV mutagenesis at 60 J m 2 was very low, but  **mutH**  or mutL or  **mutS**  mutations increased reversion of both lacZ alleles roughly 25 fold, to 5 10 x 10 8.  Ref: 10655206 Genetics, 2000
- As compared to native  **MutS** , the ability of MutS800 to promote MutL dependent activation of  **MutH**  is substantially reduced.  Ref: 12829697 J Biol Chem< MedlineTA>J Biol Chem, 2000
- **MutS** , MutL and  **MutH** .  Ref: 9852252 Int J Mol Med, 1998
- The mammalian mismatch repair system consists of homologues of the bacterial  **MutH** , MutL, and  **MutS**  proteins.  Ref: 10684825 Biol Reprod, 2000
- Such deletions are also found with starved bacteria defective in methyl directed mismatch correction activity  **mutH** , mutL or  **mutS** , and deletion mutations are also found among the much lower number of mutants that arise in bacteria wild type for mismatch correction.  Ref: 9214650 EMBO J, 1997
- This pathway is mediated by three gene products,  **MutS** , MutL, and  **MutH** , which are conserved in higher eukaryotes.  Ref: 9168943 Biochem Biophys Res Commun, 1997
- In bacteria growing exponentially in enriched minimal salts glucose medium, about 113 MutL dimers, 186  **MutS**  dimers, and 135  **MutH**  monomers were present per cell.  Ref: 8636043 J Bacteriol, 1996
- Strand specificity and repair efficiency are largely independent of the GATC methylation state of the DNA and presence of the products of mismatch repair genes  **mutH** , mutL, and  **mutS** .  Ref: 12692132 J Biol Chem< MedlineTA>J Biol Chem, 1996
- **MutH** , MutL and  **MutS**  are essential components of the mismatch repair system in Escherichia coli.  Ref: 11934505 FEMS Microbiol Lett, 2002
- Mismatch repair in many organisms depends on three proteins the mismatch recognition protein  **MutS** , a nicking endonuclease  **MutH** , and MutL, which acts as a scaffold between these.  Ref: 10973051 Trends Biochem Sci, 2000
- That mismatch repair might be down regulated in stationary phase Escherichia coli was suggested by the sequence spectrum of some stationary phase adaptive mutations and by the observations that  **MutS**  and  **MutH**  levels decline during stationary phase.  Ref: 9308969 Genes Dev, 1997
- The rate and extent of mismatch provoked cleavage at hemimethylated GATC sites by  **MutH**  in the presence of  **MutS** , MutL, and ATP are the same whether the mismatch and GATC sites are in cis or in trans.  Ref: 11371566 J Biol Chem, 2001
- In addition, normal UVM induction was observed in cells overexpressing  **MutH** , MutL, or  **MutS** .  Ref: 8955278 J Bacteriol, 1996
- However, addition of  **mutS** , mutL, or  **mutH**  on multicopy plasmids has a significant effect mutL or  **mutH**  decreases the number of mutations, while  **mutS**  stimulates mutagenesis.  Ref: 9324251 J Bacteriol, 1997
- We report here that mutations in hfq and rpoS reversed the stationary phase down regulation of the amounts of  **MutS**  and  **MutH** . hfq regulation of the amount of  **MutS**  in stationary phase cultures was mediated by RpoS dependent and independent mechanisms.  Ref: 9393714 J Bacteriol, 1997
- Furthermore, MutL greatly stimulated the d GATC specific endonuclease activity of  **MutH**  in the absence of  **MutS**  and a mispaired base.  Ref: 9880500 J Biol Chem, 1999
- The MutL,  **MutS** , and  **MutH**  proteins mediate methyl directed mismatch MDM repair and help to maintain chromosome stability in Escherichia coli.  Ref: 8636043 J Bacteriol, 1996
- After denaturation and reannealing, the PCR product pool is subjected to  **MutH** , MutL, and  **MutS**  mismatch repair proteins under double strand cleavage conditions, followed by isolation of uncleaved product by size selection.  Ref: 9192654 Proc Natl Acad Sci U S A, 1997
- Heteroduplex DNA lacking d GATC methylation is subject to mismatch provoked double strand cleavage at d GATC sites in a reaction dependent on  **MutH** , MutL,  **MutS** , and ATP.  Ref: 9192654 Proc Natl Acad Sci U S A, 1997
- These results imply that  **MutS**  and  **MutH**  decline to levels appropriate for the decreased DNA synthesis in stationary phase.  Ref: 9308969 Genes Dev, 1997
- At a low dose, dP is much more mutagenic in mismatch repair defective  **mutH** , mutL, and  **mutS**  strains than in a wild type strain.  Ref: 12196386 Genetics, 2002
- To investigate the effects on PV rates of mutations in genes for mismatch repair MMR, insertion deletion mutations of  **mutS** , mutL,  **mutH** , dam, polI, uvrD, mfd and recA were constructed in Hi strain Rd.  Ref: 11889052 EMBO J, 2002
- We also show that interaction between Vsr and MutL inhibits the ability of MutL to dimerize, to interact with  **MutS**  and  **MutH**  and to mediate a previously unknown interaction between  **MutS**  and  **MutH** .  Ref: 11585365 Mutat Res, 2001
- **MutS**  overproduction has no such effect, and MutL overproduction does not prevent stationary phase decline of either  **MutS**  or  **MutH** .  Ref: 9308969 Genes Dev, 1997
- **MutS** , MutL and  **MutH**  are the three essential proteins for initiation of methyl directed DNA mismatch repair to correct mistakes made during DNA replication in Escherichia coli.  Ref: 9482749 EMBO J, 1998
- The human mismatch repair hMMR system consists of members of three super families, namely  **MutH** , MutL and  **MutS** .  Ref: 12578652 Zhongguo Shi Yan Xue Ye Xue Za Zhi< MedlineTA>Zhongguo Shi Yan Xue Ye Xue Za Zhi, 1998
- In vitro and in vivo studies of  **MutS** , MutL and  **MutH**  mutants correlation of mismatch repair and DNA recombination.  Ref: 12606120 DNA Repair Amst , 2003
- It forms a complex on heteroduplex DNA with the mismatch recognition  **MutS**  protein and with  **MutH** , which has latent endonuclease activity.  Ref: 8692687 Nucleic Acids Res, 1996
- If heterologies are present between repeated sequences, the mismatch repair system dependent on  **MutS**  and  **MutH**  aborts potential deletion events via both mechanisms.  Ref: 9882661 J Bacteriol, 1999
- Mutation detection with  **MutH** , MutL, and  **MutS**  mismatch repair proteins.  Ref: 8633074 Proc Natl Acad Sci U S A, 1996
- The increases in spontaneous reversion of histidine mutations were first measured in isogenic strains carrying mismatch repair defective  **mutH** , mutL,  **mutS** , or uvrD alleles, relative to their mismatch repair proficient counterparts.  Ref: 9685594 Mutat Res, 1998
- Escherichia coli methyl directed mismatch repair is initiated by  **MutS** , MutL, and ATP dependent activation of  **MutH**  endonuclease, which cleaves at d GATC sites in the vicinity of a mismatch.  Ref: 8633074 Proc Natl Acad Sci U S A, 1996
- These results suggest that the amounts of  **MutS**  and  **MutH**  may be adjusted in cells subjected to different stress conditions by an RpoS dependent mechanism.  Ref: 9393714 J Bacteriol, 1997
- As judged by protein affinity chromatography, MutL and MutL E32K both support formation of ternary complexes that also contain  **MutS**  and  **MutH**  or  **MutS**  and DNA helicase II.  Ref: 10734142 J Biol Chem, 2000
- Negative regulation of  **mutS**  and  **mutH**  repair gene expression by the Hfq and RpoS global regulators of Escherichia coli K 12.  Ref: 9393714 J Bacteriol, 1997
- By in vitro comparison of MutL E32K with the wild type protein, we show the mutant protein to be defective in DNA activated ATP hydrolysis, as well as  **MutS**  and MutL dependent activation of the  **MutH**  d GATC endonuclease and the mismatch repair excision system.  Ref: 10734142 J Biol Chem, 2000
- These findings imply that the MutL nucleotide binding center is required for mismatch repair and suggest that the dominant negative behavior of the MutL E32K mutation is due to the formation of dead end complexes in which the MutL E32K protein is unable to transduce a signal from  **MutS**  that otherwise results in mismatch dependent activation of the  **MutH**  d GATC endonuclease or the unwinding activity of helicase II.  Ref: 10734142 J Biol Chem, 2000
- The in vitro activity was also dependent on products of  **mutH** , mutL,  **mutS** , and uvrD loci and was equally efficient on nucleotide insertions and deletions.  Ref: 9278429 J Biol Chem, 1997
- Consistent with this interpretation, the amount of  **MutS**  but not  **MutH**  was regulated by Hfq, but not RpoS, in exponentially growing cells.  Ref: 9393714 J Bacteriol, 1997
- We previously reported that the cellular amounts of  **MutS**  and  **MutH**  decreased by as much as 10 fold in stationary phase cultures.  Ref: 9393714 J Bacteriol, 1997
- RNase T2 protection assays showed that the amounts of  **mutS** ,  **mutH** , and mutL, but not miaA, transcripts decreased to undetectable levels in late stationary phase cells.  Ref: 8636043 J Bacteriol, 1996
- Alteration of residues in either the mismatch recognition domain, the ATPase active site, or the domain interfaces linking the two activities can diminish the differential binding of  **MutS**  to homoduplex versus heteroduplex and results in the loss of mismatch specific  **MutH**  activation.  Ref: 12606120 DNA Repair Amst , 2003
- Depletion of the cellular amounts of the  **MutS**  and  **MutH**  methyl directed mismatch repair proteins in stationary phase Escherichia coli K 12 cells.  Ref: 8636043 J Bacteriol, 1996
- The  **MutS** , MutL, and  **MutH**  proteins play major roles in several DNA repair pathways.  Ref: 9393714 J Bacteriol, 1997
- Whereas  **mutS**  and mutL genes are found in most organisms, the  **mutH**  gene is limited to some proteobacteria.  Ref: 11934505 FEMS Microbiol Lett, 2002
- We have used the recently determined crystal structures of Escherichia coli E. coli  **MutS** , MutL and  **MutH**  to guide construction of 47 amino acid substitutions in these proteins and analyzed their behavior in mismatch repair and recombination in vitro and in vivo.  Ref: 12606120 DNA Repair Amst , 2003
- We have adapted the Escherichia coli mismatch detection system, employing the factors  **MutS** , MutL and  **MutH** , for use in PCR based, automated, high throughput genotyping and mutation detection of genomic DNA.  Ref: 11222761 Nucleic Acids Res, 2001
- F lacZ targets were mated from mut donors into  **mutH** , mutL, or  **mutS**  recipients, once cells were at substantial densities, to minimize spontaneous mutation prior to irradiation.  Ref: 10655206 Genetics, 2000
- In Test A, repair proficient strain and its uvrA counterpart are used for detection of spontaneous and UV induced mutations, while in Test B mismatch repair deficient strains  **mutH** ,  **mutS** , mutL and uvrD are used for amplified detection of spontaneous mutations caused by replication errors.  Ref: 9675243 Mutat Res, 1998
- The growth defect and filamentation phenotypes associated with 2 aminopurine exposure are effectively suppressed by null mutations in  **mutH** , mutL,  **mutS** , or uvrD mutU, which encode activities that act upstream of the four exonucleases in the mechanism for the methyl directed reaction that has been proposed based on in vitro studies.  Ref: 11381137 Proc Natl Acad Sci U S A, 2001
- This phenotype is suppressed by a uvrD mutU defect, and partially suppressed by  **mutH** , mutL, or  **mutS**  mutations.  Ref: 11381137 Proc Natl Acad Sci U S A, 2001
- SOS induction was not observed in uvrC and  **mutH**  or  **mutS**  mutants defective, respectively, in excision repair and mismatch repair.  Ref: 10809694 J Bacteriol, 2000
- these frequencies were consistently 2 fold higher in  **mutH** , mutL, or  **mutS**  recipients.  Ref: 10655206 Genetics, 2000
- **mutS**  and  **mutH**  are disrupted in mutants 3 11 and 12 23, and ssaQ is disrupted in mutant 17 21.  Ref: 12654850 Infect Immun, 2003
